# Secular Trends in Energy and Macronutrient Intakes and Distribution among Adult Females (1991–2015): Results from the China Health and Nutrition Survey

**DOI:** 10.3390/nu10020115

**Published:** 2018-01-24

**Authors:** Jian Zhao, Chang Su, Huijun Wang, Zhihong Wang, Yun Wang, Bing Zhang

**Affiliations:** National Institute for Nutrition and Health, Chinese Center for Disease Control and Prevention, Beijing 100050, China; zhaojian131023@163.com (J.Z.); suchang@ninh.chinacdc.cn (C.S.); wanghj@ninh.chinacdc.cn (H.W.); wangzh@ninh.chinacdc.cn (Z.W.); wangyun@ninh.chinacdc.cn (Y.W.)

**Keywords:** female, nutrition transition, macronutrient, dietary reference intake

## Abstract

With rapid nutrition transition in China, dietary intake and nutritional status of women has gained more and more attention in the past decades. This study aimed to investigate temporal trends of total energy and macronutrient intakes among Chinese adult females. The longitudinal data are from the Chinese Health and Nutrition Survey (CHNS, 1991–2015). Information on the intake of energy and macronutrient was obtained from consecutive three-day dietary recall techniques and compared with the Chinese Dietary Reference Intakes (DRI). Mixed-effect models were performed to evaluate temporal trends of total energy and macronutrient intake. From 1991 to 2015, a significant reduction in daily energy, protein and carbohydrate intakes was seen among all adult females (*p* < 0.001). Daily fat intake, the proportion of energy from fat, the proportion of females consuming more than 30% of energy from fat and less than 50% of energy from carbohydrate were observed significant increment in the present study (*p* < 0.001). In 2015, the proportion met the DRI for energy and protein intakes were 47.0% and 48.0%, respectively; the proportion with lower carbohydrate and higher fat intakes compare with the DRI were 45.5% and 66.9%, respectively. Further nutritional education and policy interventions still needed to improve nutrition status for Chinese females.

## 1. Introduction

Over the past two decades, China has been undergoing remarkable nutrition transition and concurrent shifts in disease patterns with rapid economic growth [1,2,3,4]. The epidemic of nutrition related non-communicable diseases (NR-NCDs) including obesity, type 2 diabetes mellitus, cardiovascular diseases (CVDs), and certain cancers are continuing to challenge the health sectors in Asia [5,6,7,8,9]. As the biggest developing country, about 49% of China’s population is female. Women being vulnerable section, the impact of nutrition intake on their health is much higher [10]. It has been reported in developing countries, women have higher rates of overweight and obesity and this relationship persists over time [11]. According to the Chinese chronic disease surveillance, the prevalence of overweight and obesity of adult females were rising rapidly in recent years and up to 29.9% and 11.9%, respectively. Therefore, it is meaningful to assess the dietary intake of Chinese females and compare it to the participants of the same age group in the Dietary Reference Intakes (DRI). Furthermore, with repeated measurements over time it is possible to detect temporal changes in dietary intake.

National diet and nutrition surveys provide valuable information on a possible partial explanation for the people’s health status and disease risk [12]. In developing countries, this has been emphasized subject to many studies on children and the elderly, but ignored adult females [13,14,15]. Recently, increasing number of women are participating in the labor market. But still, women are, at large, responsible for household food preparation. In developing countries, with the population structure and economic transformation, media, fashion and other cultural influences on the nutrition transition become more prominent. Current consumption patterns of Chinese people seem to be converging towards a western diet [16]. Women may be more likely to accept these cultural influences, and the effect of culture is attached great importance to slim appearance. The desire to lean often leads to dieting or refusal to hunger, which can lead to emotional eating [17,18]. The increase in external and emotional eating is often associated with overeating and increased obesity [19,20]. Several studies on dietary patterns have shown the associations of obesity with specific dietary patterns among women, although the results are not consistent [21,22,23]. Nutrients intake was associated with reproductive hormone concentrations and differences between macronutrient intake and menstrual cycle phase [24,25,26], thus, it is important to consider women’s age-specific data when examining relationships between eating behaviors and health status.

Each of the macronutrients, carbohydrate, protein and fat, has a unique set of properties that influence health, but all are a source of energy. The optimal balance of their contribution to the diet has been a long-standing matter of debate [27]. Epidemiological studies suggest that carbohydrate intake and protein intake are inversely associated with body-mass index (BMI), while fat intake is positively associated with BMI [28,29,30]. However, studies examining trends in macronutrient intake over time have shown inconsistent results. Using longitudinal data from the China Health and Nutrition Survey (1991–2015, CHNS), the objective of this study was to analyze the trends in daily energy and macronutrient intakes among Chinese females and to investigate whether the macronutrient intake levels met the dietary reference intakes (DRI) [31]. The paper attempts to shed some light on the determinant food habit and nutritional status of the adult women in China concentrating the socio-economic status and BMI by the interpretation of a descriptive study related with nutritional status of the women.

## 2. Materials and Methods

### 2.1. Study Population

We used the data of the China Health and Nutrition Survey (CHNS) for the present investigation, which was designed to examine how the social and economic transformation in China has affected the health and nutritional status of the Chinese population [32]. The CHNS is an ongoing prospective study initiated in 1989 and have been followed up every 2–4 years, which involves twelve provinces that vary in demography, geography, economic development and public resources. Based on the level of economic development, China can be divided into four major regions (East Coast, Central China, Northeast China and Western China) to improve regional coordination and interactions so as to form a proper regional development pattern. The twelve provinces selected in the CHNS are distributed in these four regions, i.e., Heilongjiang, Liaoning and Beijing in Northeast China; Shandong, Jiangsu and Shanghai in the East Coast; Henan, Hubei, and Hunan in Central China; and Guangxi Guizhou and Chongqing in Western China. A multistage, random cluster approach was used to draw the sample surveyed in each of the provinces, and the sampling scheme is reported in details elsewhere [33]. The present analysis was based on nine rounds of survey data between 1991 and 2015, as the 1989 survey only consisted of young adults aged 20–45 years old. Our analysis consisted of 40,088 females aged 18–64 years with complete data on demographic, socioeconomic status and three-day, 24 h dietary recalls in a survey year, and, we excluded participants who were pregnant, lactating, and implausible energy intakes (<600 kcal or >4000 kcal) from the analysis. Written informed consent was provided by each participant.

This study was approved by the institutional review boards of the University of North Carolina at Chapel Hill and the National Institute for Nutrition and Health, Chinese Center for Disease Control and Prevention (2015017).

### 2.2. Dietary Data

Dietary intake at the individual level was assessed by using three consecutive 24-h dietary recalls (two weekdays and one weekend day) in each wave of the CHNS [34]. The participants were asked to report all kinds and amount of the food and beverage items (measured in grams) consumed at home and away from home during a 24-h period [35]. Interviewers were trained on using standard forms for administering the 24-h dietary recalls with food models and picture aids in the household interview. The mean daily energy intake (kcal), carbohydrate intake (g), fat intake (g) and protein intake (g) values were obtained from the dietary intake data collected in the CHNS, which were derived from the Chinese food composition table [36].

Chinese dietary reference intake standards (DRI) have been used as a standard to determine the recommended dietary intake levels for Chinese people. According to the DRI (2013) for Chinese female adults, the adequate intake of fat, carbohydrate and protein should account for 20–30%, 55–65%, and 15% of total energy, respectively [37].

### 2.3. Anthropometrics and Overweight/Obesity Status

The body weight and height of each participate were measured by well-trained health workers following standardized procedures (SECA 880 scales and SECA 206 wall-mounted metal tape). The BMI was calculated by dividing the weight (kg) by the square of height (m^2^) of each participate [38]. Accordance with the National Health and Family Planning Commission of the People’s Republic of China, the participates were defined as underweight if the BMI was below 18.5 kg/m^2^, normal if the BMI ranged between 18.5 kg/m^2^ and 24.0 kg/m^2^, and we used the cut off values of 24 kg/m^2^ and 28 kg/m^2^ to determine overweight and obesity, respectively.

### 2.4. Socio-Demographic Data

According to the age distribution of women with childbearing age and the characteristics of this data, age in years was grouped into two categories (18–49 and 49–64 years). Educational attainment was classified as: primary/illiterate, middle school, high/above. The per-capital annual income in each survey was inflated to values in 2011 by adjusting for consumer price index and then categorized into tertiles as low, medium, and high level. Geographical regions were divided into rural and urban.

### 2.5. Statistical Analysis

Statistical analyses were performed using the SAS 9.4 (SAS Institute, Inc., Cary, NC, USA). Data were subdivided according to different demographic characteristics. Adjusted means and standard errors were used to describe the distributions of continuous variables, after adjusting for complex sampling and covariates including age, education level, income level, and area of residence. Adjusted age, education level, income level and geographical region as categorical variables. The chi-square test was used to assess the association between the different levels (below, meeting and above the recommendations) of DRI for macronutrient. Mixed-effect models were performed to calculate adjusted mean intakes of total energy, macronutrient and macronutrient-energy percentages and to explore the temporal trends after adjusting for intra-class correlation within clusters and covariates.

## 3. Results

### 3.1. Social-Demographic Profile

**Figure 1:**
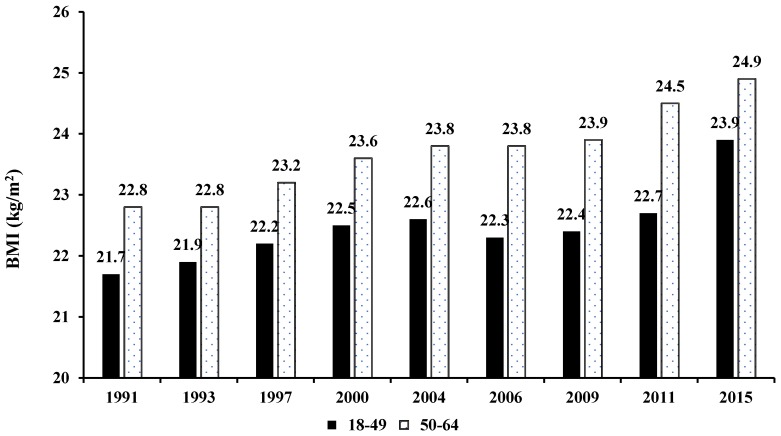
BMI changes in Chinese adult females from 1991 to 2015 by age group (18–49 and 50–64 years).

Social-demographic profiles and BMI categories of adult females aged 18–64 years in the CHNS were presented in Table 1. The sample size for the analysis was 3873 in 1991, 3856 in 1993, 3665 in 1997, 4067 in 2000, 3886 in 2004, 4545 in 2006, 4660 in 2009, 5622 in 2011and 5937 in 2015, respectively. There were significant temporal trends in age, education levels, income levels, geographical regions and BMI groups across the survey years (*p* < 0.001), which indicated that rapid economic growth and dramatic urbanity have occurred in the past 24 years in China (*p* < 0.001). During 1991–2015, the proportion of women aged 18–49 years decreased from 81.2 to 55.5%. Overweight/obesity, greater than primary school and living in urban areas became more prevalent over time of the total female samples (*p* < 0.001). In the same period, the BMI of adult women increased significantly (*p* < 0.001), and by 2015, the BMI of women aged 18–49 and 50–64 years were 23.9 and 24.9 kg/m^2^, respectively (Figure 1).

### 3.2. Energy Intake

As shown in Table 2, energy intake among the Chinese female steadily declined over time across all age (18–49 and 50–64 years), education levels, geographical regions, income levels, and BMI categories (*p* < 0.05). The average energy intake decreased 433.6 kcal/d from 1991 to 2015. Energy intake of women aged 18–49 years decreased 446.0 kcal/d and those aged 50–64 years decreased 334.7 kcal/d. As education levels increased, energy consumption decreased significantly (*p* < 0.05). The largest decline of 473.7 kcal/d was found in women in primary /illiterate. This decline was larger than those from the middle school (423.6 kcal/d) or high school or above (361.6 kcal/d). The decline of women in high-income (438.2 kcal/d) was larger than those in the medium (432.4 kcal/d) or high-income group (426.9 kcal/d). Women live in rural areas consumed more energy than those from urban areas (*p* < 0.05). Moreover, the decline of overweight/obesity (448.6 kcal/d) was smaller than normal weight women (451.2 kcal/d).

### 3.3. Fat Intake and Energy Contribution

**Figure 2:**
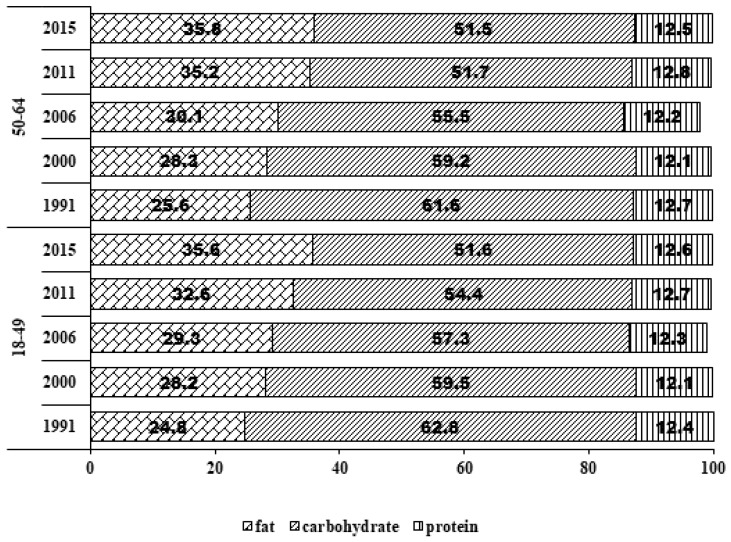
Percentage of energy from protein, fat, carbohydrate in Chinese adult females from 1991 to 2015 by age group (18–49 and 50–64 years).

**Figure 3:**
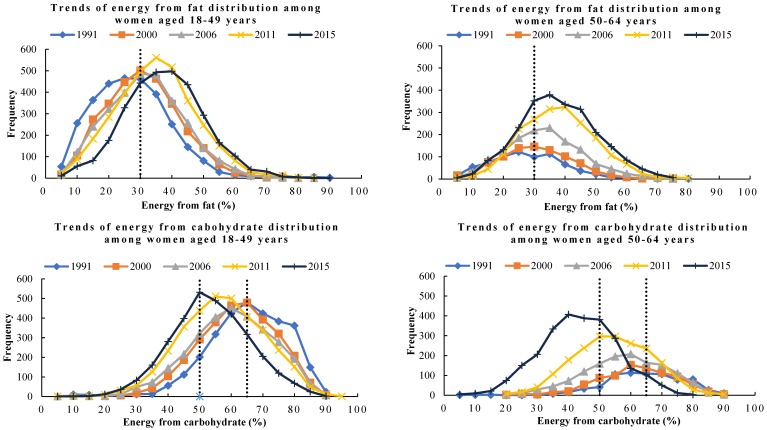
Shift in distribution of energy from fat and carbohydrate in Chinese adult females aged 18–64 years from 1991 to 2015.

Daily fat intake in Chinese adult females increased notably over time, from 62.2 g/d in 1991 to 73.6 g/d in 2015.As seen in Table 3, total fat intake increased 11.4 g/d in women aged 18–49 years and 12.0 g/d in women aged 50–64 years. The largest increase was found in women in primary/illiterate (24.7 g/d). It is worth noting that, in the highest educated population, 1991–2011 years, energy intake showed an upward trend, however, in 2011–2015, fat intake decreased significantly. The decline in fat intake in women from the low-income group was higher than those from the medium- and high-income group levels (*p* < 0.05). The distribution curves of energy from fat shifted overtime among women aged 18–49 and 50–64 years separately for selected years. As shown in Figure 2, among women aged 18–49 and 50–64 years, percentages of energy from fat increased 10.0% and 10.2%, separately. The main characteristics of the changes for both age groups were the shift in the curves to the right (Figure 3).

### 3.4. Carbohydrate Intake and Energy Contribution

Table 4 shows that daily carbohydrate intake steadily dropped across the survey years in all ages (18–49 and 50–64 years), education levels, geographical regions, income levels, geographical regions and BMI groups (*p* < 0.05). Daily carbohydrate intake dropped from 358.0 g in 1991 to 236.9 g in 2015. The decline in carbohydrate intake in women aged 18–49 years (124.7 g/d) was larger than women in 50–64 years (99.1 g/d). In addition, the largest decline in primary or illiterate women (161.0 g/d) was larger than those from the middle (111.1 g/d) or high-education group (81.4 g/d). The decline in rural women (136.5.1 g/d) was larger than those from urban areas (88.8 g/d). The decline in carbohydrate intake in women from the low-income group (136.8 g/d) was higher than that in those from the medium-(110.4 g/d) and high-income group levels (116.5 g/d). Moreover, the decline of 124.9 g/d in normal weight women was larger than that in the overweight or obesity (120.0 g/d) or underweight (98.2 g/d). As showed in Figure 3, the distribution curves of energy from carbohydrate shifted overtime among women aged 18–49 and 50–64 years separately for selected years. The main characteristics of the changes for both age groups were the shift in the curves to the left. Percentages of energy from carbohydrate decreased 11.2% in women aged 18–49 years and 11.1% in those aged 50–64 years (Figure 2).

### 3.5. Protein Intake and Energy Contribution

Table 5 presents the daily protein intake among Chinese adult females from 1991 to 2015, which steadily declined from 69.8 g to 57.0 g during this period (*p* < 0.05). We observed daily protein intake steadily dropped across the survey years in each age group, education, work, income and different regions (*p* < 0.05). The decline in protein intake in the younger age group (12.6 g/d) was larger than that in older group (11.5 g/d). The decline in low-education group (14.7 g/d) was larger than those from middle (12.9 g/d) or high-education group (9.7 g/d). The decline of women lives in rural (14.1 g/d) was larger than those from urban areas (9.6 kcal/d). Moreover, the largest decline of 14.3 g/d in protein intake was found in overweight or obesity group. This decline was larger than that in the normal weight (13.0 g/d) or underweight group (8.6 g/d). However, percentages of energy from protein maintain steady states in women of two age groups from 1991 to 2015.

### 3.6. Percentage of Females with Intake Levels Meeting the Dietary Reference Intakes

The percentage of women with relative macronutrient intake levels meeting, below and above the DRI, classified by age groups, education levels, income levels geographical regions and BMI groups of China. The proportion of women with more than 30% of energy from fat increased significantly from 31.8% in 1991 to 66.9% in 2015 (Table A1). Women aged 18–49 years increased from 31.0 to 67.1%, while women aged 50–64 years increased from 35.2 to 66.6%. The greatest increase in the proportion of women who consumed more than 30% of energy from fat was found in the primary/illiterate group (56.0%). In 2015, there were about 41.3% of adult women met the DRI. Likewise, as shown in Table A2. The proportion of women consuming a diet with less than 50% of energy from carbohydrate increased from 14.1% in 1991 to 45.5% in 2015. Women aged 18–49 years increased from 14.0 to 45.7%, while women aged 50–64 years increased from 14.3 to 45.2%. The greatest increase in the proportion of women who consumed less than 50% of energy from carbohydrate was found in the primary/illiterate group (52.5%). More than 45% of the women had relative fat intakes above the DRI by 2015.

## 4. Discussion

This is the first attempt to report daily energy and macronutrient intakes in representative female samples from nine major provinces across the country using 2015 updated CHNS data. The present study is an effort to determine the nutritional status of the Chinese adult females concentrating the social-economic status and BMI categories by the interpretation of a longitudinal descriptive study related with nutritional status. During the past decades, a significant reduction in daily energy, protein and carbohydrate intakes was seen in all categories among all adult females in China. Conversely, fat intake of the females tends to increase from 1991 to 2015. Findings from the present research indicate that Chinese adult females have been undergoing a rapid nutrition transition towards a low-carbohydrate and high-fat diet. The observed trend is consistent with that in Swedish women between 2007 and 2012 [39]. It is noteworthy in our study that the largest transition in dietary patterns took place in females from primary/illiterate education level.

Our study shows that dietary energy intake in Chinese adult females declined significantly over the past two decades. It is consistence with a previous study in Indian rural women, whose energy intake decrease steadily from 2014.0 kcal in 1997 to 1780.0 kcal in 2011 [40]. Given the rapid increase in the prevalence of overweight and obesity in Chinese women, a potential explanation for this decline in total energy intake is a decrease in total energy expenditure [41]. Large studies conducted in China, in particular using data of the CHNS, reported average physical activity by the occupational, domestic, active leisure and travel domains decreased with modernization among adult women from 1991 to 2011 [42]. Up to the highest education level, energy intake was gradually declined. This is probably associated with increased awareness of health issues associated with excess energy. These findings agree with a previous study in developed countries, which showed calorie consumption is inversely associated with education levels [43]. The low energy of high-earning women and those live in urban areas may be due to more job opportunities, service of health professionals and availability of recreational facilities that have changed those behaviors that are not conducive to controlling obesity. Meanwhile, cost is a strong influence on food purchases and given that persons of low social-economics status (SES) often have more limited budgets, healthier foods such as fruit and vegetables may be overlooked in favor of less healthy, more energy-dense options [44]. In addition, this study found that the overweight/obese subjects consumed more energy than those normal weight women, confirms that obese people need more energy to maintain their weight. This positive correlation between energy intake and body weight status is further supported by results of the WHO Multinational Monitoring of Trends and Determinants in Cardiovascular Disease (MONICA) aggregate level analyses which found that increasing energy supply is closely associated with the increase in overweight and obesity in European countries [45].

As a main source of dietary energy, carbohydrate plays a critical role in the Chinese traditional diet. The decrease in carbohydrate observed in our study can partly be explained by the drop in energy expenditure as sedentary behaviors is ubiquitous in daily lives of modern Chinese women. Previous research shows that carbohydrate might promote the development of small intestinal bacterial overgrowth in obesity [46]. Some arguing that carbohydrate intake has a more prominent role in promoting weight gain than dietary fat [47]. Our results suggest that overweight/obese individuals were reported consuming a somewhat larger fat but smaller carbohydrate, compared with underweight/normal weight individuals, indicated that increasing trends in energy consumption are of concern for all BMI classes, especially for individuals in the highest BMI classes. Moreover, urban movement has had a significant effect on the amount and type of food produced and consumed in China. For example, agriculture’s share of the economy has continued to decrease since 1970; replaced by livestock, fisheries, and other commodities. This has led to modifications in the ‘traditional’ Chinese diet; increasing the fat intake and decreasing the carbohydrates usually found in the Chinese diets.

A dramatic decline was observed regarding the consumption of total protein in adult female diets, supported by a previous study which showed a slow decrease in protein intake in women between 1997 and 2011. The reasons for the decline in protein intake are difficult to identify and can only be speculated. For instance, a potential explanation may be due to significantly reduced plant sources (rice and pulses) food. Future efforts are needed to explore the other impassible reasons. Our study indicate overweight/obesity females intake the most protein, consistent with the results of a systematic review and meta-analysis, conducted to assess the benefits and harms of higher-protein compared with lower-protein diets in the general population and find that higher-protein diets probably improve adiposity [48].

The present study indicates that the total energy intakes derived from fat, carbohydrate and protein among females in the 2015 CHNS were 35.7%, 51.6% and 12.6%, respectively. The Chinese dietary guideline (CDG) of 2016 version suggested for Chinese adults that the total energy derived from fat, carbohydrate and protein were below 30%, 55–65% and 10–15%, respectively. The proportion of total energy from protein is in line with the CDG recommendation, the proportion of total energy from carbohydrate below the recommendation. While the energy from fat exceeded the recommendation. Moreover, the proportion of women consuming a diet with more than 30% of total energy from fat more than doubled from 1991 to 2015 (nearly 67%). Excessive fat intake may lead to the development of NCD and chronic condition. Accordingly, the Chinese government need to take immediate actions to carry out effective interventions to promote a healthy diet for Chinese women, considering a rapid acceleration of nutrition and epidemiological transition with increased burden of NCD.

The global prevalence of overweight and obesity as a public health concern is well established and reflects the overall worldwide lack of success in achieving and maintaining a healthy body weight [49]. Increasing evidence showed that the prevalence of overweight and obesity has increased dramatically over the past decades in China [50]. In contrast, the importance of gender as a variable in obesity is less well appreciated. Obesity may cause greater harms to females, lead to reproductive endocrine disorders, breast cancer, menstrual disorders, abortion, and so on. Our study found time trends in energy and macronutrient intakes were similar across all three BMI categories. From1991 to 2015, overweight or obesity rates in China’s adult females have risen steadily, with the average rate at 45.4% in 2015. It can be predicted that the burden of diseases caused by obesity of female will increase greatly in China in the future. There for women themselves should have a balanced dietary pattern and healthy lifestyle which they can hold up as an example to their children, which would help prevent the vicious cycle of inter-generational obesity. Efforts should be made to help Chinese women to develop a healthy lifestyle and adopt healthy dietary habits from an early age.

It is worth our attention that by 2015, total energy intake and energy composition of adult women in two age groups is basically consistent. However, the average BMI of women aged 50–64 is significantly higher than that of women aged 18–49. Our research results indicated that overweight and obese women might closely related to energy consumption. Growth with age, activities and hormone levels of women usually significant changes. The relationships between the energy consumption difference and overweight and obesity among women in different age groups will be further discussed in our next research.

Major strengths of this study include the use of the longitudinal data (CHNS) with the latest (2015) and large sample size. The use of individual, consecutive three-day recall method could improve the accuracy of dietary recalls and hence the analysis and results. Moreover, examining this issue in prospective studies that repeatedly collect both dietary intake and body weight data may provide further insight. Our results also highlight several other demographic and lifestyle trends in the Chinese females over the covered time period.

In evaluation of the results, mentioning some limitations seems necessary. As with other population research, dietary data were collected using three consecutive 24-h dietary recalls, which might have relatively limited corrections for within-subject variations compared to non-consecutive 24-h-recalls. However, the average intake over three days can offer a relatively valid estimate of nutrient intakes, as shown in an earlier study using the CHNS. Moreover, macronutrient groups exist in different foods, pointing out to these imbalances makes it impossible to consider which food exactly causes the mentioned deficiency. In addition, the CHNS does not present national data and the vast, western areas of China were not included in the present study.

## 5. Conclusions

Chinese adult females have been undergoing a rapid nutrition transition towards a high-fat and low-carbohydrate diet. Though the overall dietary condition of women is improving in developing countries like China, potential problems of Chinese rapid nutrition transition still existed, especially in those from low-education and low-income level. Public health policies should pay more attention to the excess of total fat intake and higher proportion of energy from total fat. Further nutritional education and intervention are still needed to ensure a more balanced diet for adult females in China.

## Figures and Tables

**Table 1:** Characteristics of participants aged 18–64 years from 1991 to 2015 in China Health and Nutrition Surveys ***^,†^.

General Characteristics	1991	1993	1997	2000	2004	2006	2009	2011	2015
Sample (*n*)	3873	3586	3665	4067	3886	4545	4660	5622	5937
Age groups (years)									
18–49 (%)	81.2	83.6	80.7	78.5	69.6	70.0	67.2	65.0	55.5
50–64 (%)	18.8	16.4	19.3	21.5	30.4	30.0	32.8	35.0	43.1
Education level									
Primary/illiterate (%)	56.9	53.7	52.3	45.4	43.0	39.3	39.8	34.6	27.5
Middle school (%)	27.9	30.3	28.8	32.4	32.8	34.8	36.3	34.7	33.7
High/above (%)	15.2	16.0	19.0	22.2	24.2	25.9	23.8	30.7	36.2
Income									
Low (%)	31.1	33.4	33.3	33.3	33.3	33.3	33.3	33.3	31.0
Medium (%)	35.6	33.3	33.4	33.4	33.4	33.3	33.3	33.4	33.2
High (%)	33.3	33.4	33.3	33.3	33.3	33.3	33.3	33.3	35.8
Region									
Rural (%)	66.8	68.5	67.3	67.3	67.5	70.1	71.4	65.9	61.1
Urban (%)	33.2	31.5	32.7	32.7	32.5	29.9	28.6	34.1	38.9
BMI category									
Underweight (%)	8.6	8.3	7.4	6.7	6.3	6.1	6.8	6.0	6.2
Normal (%)	69.8	68.2	65.5	61.0	58.7	61.5	59.4	56.4	48.4
Overweight/obesity (%)	21.6	23.5	27.1	32.4	35.0	32.4	33.7	37.6	45.4

*** Significant trend in each subgroup across the survey years (*p* < 0.001; test for trend); ^†^ Values adjusted for age, education, income, region and body-mass index (BMI).

**Table 2:** Daily energy intake (kcal), by age, education, income and geographical region in Chinese women from 1991 to 2015 ^1, 2^.

	1991		1993		1997		2000		2004		2006		2009		2011		2015	
	Mean	SE	Mean	SE	Mean	SE	Mean	SE	Mean	SE	Mean	SE	Mean	SE	Mean	SE	Mean	SE
**Age group (year)**																		
18–49	2300.7	11.0	2255.1	11.2	2286.4	11.1	2183.7	10.5	2133.6	11.5	2142.0	10.9	2080.9	10.7	1952.7	10.0	1854.7	10.3
50–64	2158.8	20.8	2187.6	23.5	2176.9	22.5	2122.7	20.1	2137.1	18.0	2138.0	16.8	2025.3	14.8	1877.7	13.2	1824.1	11.4
**Education level**																		
Primary/illiterate	2307.5	13.3	2291.9	14.0	2302.9	14.0	2207.1	13.9	2188.7	15.0	2211.3	15.0	2125.7	14.2	1970.2	14.0	1835.8	14.3
Middle school	2251.5	18.3	2210.4	18.1	2249.3	18.2	2177.8	16.5	2105.0	17.1	2155.7	15.0	2069.9	14.6	1963.0	13.6	1847.2	14.6
High/above	2189.5	23.4	2147.4	23.8	2185.7	22.5	2085.6	19.0	2079.0	18.9	2013.6	17.4	1946.5	16.2	1835.8	13.6	1840.4	11.6
**Income**																		
Low	2294.7	18.5	2282.4	18.6	2288.6	7.1	2196.0	16.4	2110.5	16.7	2200.7	16.4	2123.1	15.8	2014.1	14.2	1867.8	14.2
Medium	2266.4	16.2	2225.0	16.5	2264.0	17.2	2174.3	16.0	2190.2	16.9	2143.6	5.4	2046.3	14.9	1907.9	13.9	1834.0	13.4
High	2262.7	16.4	2224.6	17.3	2243.2	17.5	2141.5	16.0	2103.2	16.9	2078.2	15.6	2018.6	14.3	1857.4	13.2	1824.5	12.3
**Region**																		
Rural	2312.4	12.4	2268.2	12.4	2270.0	12.1	2196.8	11.3	2160.2	11.6	2180.0	10.9	2117.0	10.4	2001.7	9.9	1845.3	10.0
Urban	2196.6	15.7	2191.5	17.2	2255.6	17.6	2116.6	16.3	2081.7	17.6	2048.9	16.6	1927.3	15.4	1781.2	12.8	1834.5	11.9
**BMI category**																		
Underweight	2149.0	33.4	2188.3	34.4	2242.6	35.4	2134.6	36.8	2107.6	40.6	2071.7	35.7	1950.3	31.7	1929.0	32.0	1840.1	30.1
Normal	2280.8	11.8	2243.8	12.4	2278.8	12.5	2187.6	12.0	2134.6	12.7	2149.7	11.9	2081.1	11.6	1946.2	10.7	1829.6	10.9
Overweight/obesity	2302.1	20.6	2264.2	19.8	2238.8	18.8	2146.0	16.0	2139.6	16.4	2136.8	15.6	2053.2	14.4	1896.5	12.9	1853.5	11.4
**Total**	2274.0	9.8	2244.0	10.1	2265.3	10.0	2170.6	9.3	2134.7	9.7	2140.8	9.1	2062.7	8.7	1926.5	8.0	1841.1	7.6

^1^ Significant trend in each subgroup across the survey years (*p* < 0.001; test for trend); ^2^ Values adjusted for age, education, income, region and BMI. SE, Standard Error.

**Table 3:** Daily fat intake (g), by age, education, income and geographical region, in Chinese women from 1991 to 2015 ^1,2^.

	1991		1993		1997		1997		2000		2004		2006		2009		2011		2015	
	Mean	SE	Mean	SE	Mean	SE	Mean	SE	Mean	SE	Mean	SE	Mean	SE	Mean	SE	Mean	SE	Mean	SE
**Age group (year)**																				
18–49	62.4	0.6	61.8	0.6	67.2	0.7	67.2	0.7	69.6	0.7	68.6	0.7	70.2	0.7	69.0	0.6	70.4	0.6	73.8	0.6
50–64	61.2	1.2	66.1	1.5	69.3	1.5	69.3	1.5	68.3	1.3	67.0	1.1	75.1	1.2	73.0	0.9	73.8	0.8	73.2	0.7
**Education level**																				
Primary/illiterate	57.2	0.7	57.7	0.8	62.0	0.8	62.0	0.8	65.4	0.9	63.6	0.9	67.9	0.9	69.4	0.9	68.7	0.8	81.9	0.9
Middle school	67.0	1.1	64.6	1.0	70.0	1.1	70.0	1.1	70.3	1.0	67.5	1.1	71.5	1.0	68.7	0.9	70.3	0.8	76.5	0.9
High/above	71.8	1.4	74.7	1.7	79.6	1.5	79.6	1.5	76.1	1.3	77.0	1.3	77.6	1.2	74.2	1.0	76.2	0.8	66.5	0.7
**Income**																				
Low	54.6	0.9	56.0	1.0	59.3	0.9	59.3	0.9	62.0	1.0	58.2	1.0	64.9	1.0	65.0	1.0	65.4	0.8	73.6	0.9
Medium	63.4	0.9	63.4	1.0	67.9	1.1	67.9	1.1	70.4	1.0	70.4	1.1	71.2	1.0	71.0	0.9	73.4	0.8	70.6	0.8
High	67.9	1.0	68.3	1.1	75.8	1.1	75.8	1.1	75.6	1.1	75.7	1.1	78.9	1.1	74.9	0.8	75.9	0.8	76.2	0.8
**Region**																				
Rural	58.1	0.7	56.8	0.7	61.2	0.7	61.2	0.7	64.7	0.7	63.9	0.7	67.7	0.7	68.5	0.6	69.6	0.6	73.3	0.6
Urban	70.3	1.0	75.0	1.1	80.9	1.2	80.9	1.2	79.0	1.1	76.9	1.2	80.9	1.2	74.8	1.0	75.4	0.8	74.0	0.7
**BMI category**																				
Underweight	59.3	2.0	60.9	2.0	68.9	2.5	68.9	2.5	66.7	2.3	65.3	2.7	67.9	2.3	64.6	1.9	69.7	2.0	72.5	1.9
Normal	61.8	0.7	61.6	0.7	66.7	0.8	66.7	0.8	68.4	0.7	67.2	0.8	71.4	0.8	70.3	0.7	71.1	0.7	72.9	0.7
Overweight/obesity	64.6	1.2	65.9	1.3	69.7	1.2	69.7	1.2	71.6	1.1	70.3	1.0	72.8	1.0	71.5	0.9	72.6	0.8	74.4	0.7
**Total**	62.2	0.6	62.5	0.6	67.7	0.6	67.7	0.6	69.3	0.6	68.1	0.6	71.7	0.6	70.3	0.5	71.6	0.5	73.6	0.5

^1^ Significant trend in each subgroup across the survey years (*p* < 0.001; test for trend); ^2^ Values adjusted for age, education, income, region and BMI.

**Table 4:** Daily carbohydrate intake (g), by age, education, work status, income and geographical region, in Chinese women from 1991 to 2015 ^1,2^.

	1991		1993		1997		2000		2004		2006		2009		2011		2015	
	Mean	SE	Mean	SE	Mean	SE	Mean	SE	Mean	SE	Mean	SE	Mean	SE	Mean	SE	Mean	SE
**Age group (year)**																		
18–49	363.8	2.2	353.9	2.2	351.4	2.1	322.8	1.8	311.7	2.0	306.3	2.0	295.3	2.0	267.4	1.8	239.1	1.7
50–64	333.3	4.1	331.1	4.4	323.8	4.0	311.3	3.4	315.8	3.2	293.6	2.9	273.5	2.5	243.2	2.2	234.2	1.9
**Education level**																		
Primary/illiterate	377.6	2.8	373.3	2.9	369.4	2.7	340.0	2.5	337.3	2.7	329.0	2.9	308.2	2.6	278.5	2.6	216.6	2.2
Middle school	341.6	3.4	336.8	3.3	335.9	3.3	319.0	2.8	308.0	2.9	307.8	2.6	293.4	2.6	270.1	2.4	230.5	2.3
High/above	315.1	4.0	298.1	3.9	297.5	3.7	281.9	2.9	276.4	3.1	255.1	2.5	246.5	2.5	224.1	2.1	233.7	2.0
**Income**																		
Low	381.5	3.9	375.2	3.6	372.3	3.4	344.6	3.0	331.7	3.0	334.2	3.1	319.2	3.0	294.9	2.7	244.7	2.4
Medium	353.3	3.2	344.0	3.3	344.6	3.2	319.0	2.7	320.7	3.0	303.6	2.8	282.1	2.6	250.6	2.3	242.9	2.3
High	341.1	3.1	331.3	3.2	321.4	3.1	297.3	2.7	286.4	2.8	269.7	2.5	263.1	2.4	231.2	2.1	224.6	1.9
**Region**																		
Rural	376.7	2.6	369.6	2.5	363.3	2.3	338.9	2.0	329.9	2.1	322.6	2.0	307.4	1.9	282.6	1.8	240.2	1.7
Urban	320.5	2.7	307.9	2.9	310.7	3.1	282.0	2.5	277.7	2.8	255.2	2.5	240.0	2.5	213.0	1.9	231.7	1.9
**BMI category**																		
Underweight	338.3	6.5	342.4	6.4	340.8	6.0	318.6	6.3	313.1	7.2	296.8	6.6	276.7	5.6	264.3	5.6	240.1	5.2
Normal	360.4	2.4	352.7	2.5	351.8	2.4	326.4	2.1	315.9	2.3	306.0	2.1	292.4	2.1	264.9	1.9	235.5	1.8
Overweight/obesity	358.0	4.2	345.6	3.9	333.9	3.5	309.2	2.7	307.9	2.8	296.8	2.8	283.0	2.5	249.1	2.1	238.0	1.9
**Total**	358.0	2.0	350.2	2.0	346.1	1.9	320.3	1.6	312.9	1.7	302.5	1.7	288.1	1.6	258.9	1.4	236.9	1.3

^1^ Significant trend in each subgroup across the survey years (*p* < 0.001; test for trend); ^2^ Values adjusted for age, education, income, region and BMI.

**Table 5:** Daily protein intake (g), by age, education, work status, income and geographical region, in Chinese women from 1991 to 2015 ^1,2^.

	1991		1993		1997		2000		2004		2006		2009		2011		2015	
	Mean	SE	Mean	SE	Mean	SE	Mean	SE	Mean	SE	Mean	SE	Mean	SE	Mean	SE	Mean	SE
**Age group (year)**																		
18–49	70.3	0.4	69.9	0.4	67.9	0.4	65.7	0.4	65.0	0.4	65.2	0.4	63.9	0.4	61.0	0.4	57.7	0.4
50–64	67.7	0.7	66.3	0.9	63.6	0.8	63.9	0.7	64.8	0.7	64.6	0.6	62.4	0.6	59.0	0.5	56.2	0.4
**Education level**																		
Primary/illiterate	69.6	0.5	68.9	0.5	66.0	0.5	63.7	0.5	64.1	0.5	64.5	0.5	62.7	0.5	58.0	0.5	57.0	0.5
Middle school	69.9	0.6	69.8	0.7	67.9	0.6	66.3	0.6	64.2	0.6	65.6	0.6	63.9	0.5	60.8	0.5	58.2	0.6
High/above	70.4	0.8	69.9	0.9	68.7	0.8	67.0	0.7	67.3	0.7	64.9	0.7	63.8	0.7	62.2	0.6	56.3	0.4
**Income**																		
Low	68.9	0.6	68.7	0.6	65.7	0.6	64.0	0.6	62.7	0.6	64.9	0.6	63.0	0.5	59.8	0.5	55.7	0.5
Medium	70.0	0.6	69.1	0.6	67.7	0.6	64.9	0.6	66.2	0.6	65.2	0.6	63.1	0.6	59.4	0.5	55.9	0.5
High	70.5	0.6	70.3	0.6	67.7	0.6	67.0	0.6	65.9	0.6	65.0	0.6	64.1	0.5	61.5	0.5	59.2	0.5
**Region**																		
Rural	69.6	0.5	70.4	0.6	69.7	0.6	67.8	0.6	66.8	0.6	66.6	0.7	64.0	0.6	61.3	0.5	55.4	0.3
Urban	69.9	0.4	68.8	0.4	65.8	0.4	64.1	0.4	64.1	0.4	64.3	0.4	63.2	0.4	59.7	0.3	59.5	0.4
**BMI category**																		
Underweight	64.9	1.1	67.3	1.2	63.8	1.2	63.5	1.4	63.9	1.5	62.5	1.4	59.2	1.1	59.0	1.2	56.3	1.0
Normal	69.9	0.4	68.9	0.4	66.9	0.4	65.5	0.4	64.6	0.5	64.7	0.4	63.6	0.4	60.6	0.4	57.0	0.4
Overweight/obesity	71.5	0.7	71.3	0.7	68.3	0.7	65.2	0.6	65.8	0.6	66.1	0.6	63.9	0.5	60.0	0.5	57.2	0.4
**Total**	69.8	0.3	69.3	0.4	67.1	0.3	65.3	0.3	64.9	0.4	65.0	0.3	63.4	0.3	60.3	0.3	57.0	0.3

^1^ Significant trend in each subgroup across the survey years (*p* < 0.001; test for trend); ^2^ Values adjusted for age, education, income, region and BMI.

## Appendix A

**Table A1:** Energy from fat compare to the DRIs standard in Chinese female adults from 1991 to 2015 ^2^.

	20–30% ^1^									> 30% ^1^								
	1991	1993	1997	2000	2004	2006	2009	2011	2015	1991	1993	1997	2000	2004	2006	2009	2011	2015
**Age group (year)**																		
18–49	31.8	32.9	32.5	32.7	30.7	29.9	30.1	25.8	23.6	31.0	30.6	36.2	42.0	43.7	46.8	48.7	58.0	67.1
50–64	30.7	31.5	32.5	32.9	29.5	30.5	32.3	25.1	24.9	35.2	38.3	41.6	42.2	41.9	50.9	55.2	65.9	66.6
**Education level**																		
Primary/illiterate	30.6	31.6	32.7	33.4	29.7	31.2	32.9	28.3	13.3	24.6	24.6	28.1	34.2	34.5	39.8	45.1	52.6	80.6
Middle school	32.7	34.3	33.9	33.3	32.8	30.9	30.9	27.3	18.9	37.9	35.0	40.2	43.3	43.0	46.8	48.1	56.9	73.7
High/above	33.2	33.4	29.9	30.7	27.9	27.4	27.3	20.4	34.1	47.6	50.4	58.0	56.4	58.6	62.1	64.4	74.3	54.2
**Income**																		
Low	27.8	31.4	33.0	32.6	30.8	31.5	32.5	29.8	26.2	23.3	22.7	25.4	30.7	30.2	35.8	38.6	46.4	64.2
Medium	33.1	32.2	33.9	34.0	31.5	32.6	30.6	25.5	25.8	32.5	34.5	37.3	43.3	43.9	46.6	53.3	63.4	62.5
High	33.5	34.6	30.7	31.8	28.6	26.6	29.4	21.4	20.9	39.0	38.5	49.1	52.2	55.3	61.6	60.6	72.5	73.3
**Region**																		
Rural	31.2	32.8	34.8	34.9	32.6	32.8	33.2	29.2	25.4	25.5	23.9	28.1	33.7	36.2	40.6	45.1	52.9	64.6
Urban	32.4	32.6	28.0	28.5	25.5	23.8	24.9	18.5	22.3	44.4	49.3	56.1	59.4	57.6	65.5	65.1	75.9	70.4
**BMI category**																		
Underweight	31.6	36.5	35.6	35.8	30.0	30.0	31.9	29.1	26.9	30.7	29.4	35.2	36.9	39.3	45.8	48.0	56.8	64.7
Normal	31.6	32.0	33.4	33.1	30.4	29.9	30.5	25.8	24.2	31.1	31.1	35.1	40.5	41.6	46.9	50.0	58.7	66.7
Overweight/obesity	31.5	33.5	29.5	31.5	30.2	30.6	31.2	24.6	23.8	34.6	35.1	42.8	46.1	46.3	50.4	52.8	64.4	67.4
**Total**	31.6	32.7	32.5	32.8	30.3	30.1	30.8	25.5	24.2	31.8	31.9	37.2	42.1	43.1	48.0	50.8	60.8	66.9

^1^ Significant trend in each subgroup across the survey years (*p* < 0.001; test for trend); ^2^ Values adjusted for age, education, income, region and BMI.

**Table A2:** Energy from carbohydrate compare to the DRIs standard in Chinese female adults from 1991 to 2015 ^2^.

	50–65% ^1^									< 50% ^1^								
	1991	1993	1997	2000	2004	2006	2009	2011	2015	1991	1993	1997	2000	2004	2006	2009	2011	2015
**Age group (year)**																		
18–49	41.2	41.5	42.8	44.6	42.2	43.0	44.3	41.4	40.5	14.0	14.4	16.4	21.5	24.6	28.7	30.4	33.7	45.7
50–64	44.9	42.8	44.8	45.1	43.2	42.4	48.2	40.4	42.3	14.3	18.5	20.4	22.1	21.6	32.7	34.7	45.1	45.2
**Education level**																		
Primary/illiterate	35.7	35.3	38.1	41.5	39.3	40.8	45.4	42.0	28.7	10.7	11.0	11.0	15.5	16.5	22.2	24.9	31	63.2
Middle school	48.3	47.9	47.5	46.1	45.1	44.8	46.8	42.9	38.7	16.2	14.8	18.3	22.4	21.3	26.0	29.7	31.1	51.2
High/above	53.4	51.1	50.7	49.4	44.7	43.1	43.9	37.8	50.7	22.4	29.6	32.5	32.9	38.0	44.3	46	54.4	31.0
**Income**																		
Low	33.6	34.8	38.1	40.2	38.8	40.9	44.2	44.3	43.2	9.2	9.3	9.1	13.1	13.9	18.3	19.6	24.1	42.0
Medium	44.0	41.8	44.4	48.2	44.9	45.5	46.8	41.0	41.2	14.9	16.7	17.0	20.8	22.5	27.4	31.4	42.6	41.5
High	47.5	48.5	47.1	45.8	43.9	42.0	45.7	37.7	39.7	17.6	19.2	25.3	30.9	34.5	42.3	41.8	52.2	52.2
**Region**																		
Rural	37.0	37.8	41.5	43.4	43.3	44.7	48.5	44.9	41.3	11.0	9.0	9.8	14.1	16.4	21.2	23.3	29.7	43.3
Urban	51.9	50.1	46.7	47.4	41.0	38.4	38.3	33.5	41.3	20.0	28.2	32.3	37.1	38.6	48.5	49.8	58.7	48.8
**BMI category**																		
Underweight	43.0	45.9	41.5	44.6	36.1	40.4	46.1	40.3	41.6	13.7	11.9	17.8	19.2	23.3	26.7	27.9	36.4	43.7
Normal	41.4	40.1	42.6	44.4	41.6	42.8	44.4	40.7	41.0	13.7	14.8	15.8	20.3	23.1	27.1	30.7	37.7	45.6
Overweight/obesity	43.2	44.7	45.1	45.3	45.2	43.3	47.5	41.6	41.6	15.1	17.1	20.3	24.7	24.6	31.1	32	43	45.5
**Total**	41.9	41.7	43.2	44.7	42.5	42.8	45.6	41.0	41.3	14.1	15.1	17.1	21.7	23.4	26.6	30.9	39.6	45.5

^1^ Significant trend in each subgroup across the survey years (*p* < 0.001; test for trend); ^2^ Values adjusted for age, education, income, region and BMI.
